# Long Noncoding RNA KCNQ1OT1 is a Prognostic Biomarker and mediates CD8^+^ T cell exhaustion by regulating CD155 Expression in Colorectal Cancer

**DOI:** 10.7150/ijbs.59001

**Published:** 2021-04-22

**Authors:** Zheng-bin Lin, Pei Long, Zhan Zhao, Yi-ran Zhang, Xiao-dong Chu, Xiao-xu Zhao, Hui Ding, Song-wei Huan, Yun-long Pan, Jing-hua Pan

**Affiliations:** 1 Department of General Surgery, The First Affiliated Hospital of Jinan University, Guangzhou, 510632, China.; 2 College of Pharmacy, Jinan University, Guangzhou, 510632, China.; 3 Department of Bone and Joint Surgery, The First Affiliated Hospital of Jinan University, Guangzhou, 510630, China.; 4 MOE Key Laboratory of Tumor Molecular Biology and Key Laboratory of Functional Protein Research of Guangdong Higher Education Institutes, Institute of Life and Health Engineering, Jinan University, Guangzhou, 510630, China.

**Keywords:** lncRNA KCNQ1OT1, cancer, prognosis, bioinformatics, tumor-infiltrating, T cell exhaustion

## Abstract

**Background:** Long noncoding RNA KCNQ1 opposite strand/antisense transcript 1 (lncRNA KCNQ1OT1) is abnormally expressed in various solid tumors. The purpose of this study was to explore the prognostic value and potential functional role of lncRNA KCNQ1OT1 across cancers.

**Methods:** We performed a meta-analysis of published literature to evaluate the prognostic value of lncRNA KCNQ1OT1 across cancers. Verification, functional analysis, and genomic variation analysis were performed using the GEPIA, TIMER, and LnCeVar databases. According to the immune cell infiltration level, we established a prognostic model of lncRNA KCNQ1OT1 expression using public datasets of TIMER. We used quantitative real-time polymerase chain reaction (RT-qPCR) and western blot to detect the expression levels of lncRNA KCNQ1OT1 and the CD155 protein in colorectal cancer (CRC) tissues and cell lines. Then, a lncRNA KCNQ1OT1-knockdown cell line was cocultured to explore the role of lncRNA KCNQ1OT1 and CD155 in the T cell response by flow cytometric analysis.

**Results:** Our results showed that the high expression of lncRNA KCNQ1OT1 was significantly related to poor overall survival across cancers, especially CRC. Interestingly, we found that COAD patients with high lncRNA KCNQ1OT1 expression and high CD8^+^ T cell infiltration levels had a worse prognosis than those with low lncRNA KCNQ1OT1 expression and high CD8^+^ T cell infiltration levels. Moreover, lncRNA KCNQ1OT1 and CD155 showed significantly higher expression in CRC tissue than in normal tissue, and lncRNA KCNQ1OT1 expression was positively correlated with CD155 expression in CRC. Finally, knockdown of lncRNA KCNQ1OT1 reduced CD155 expression in HCT116 and SW620 cells and enhanced the immune response in coculture with CD8^+^ T cells.

**Conclusions:** High lncRNA KCNQ1OT1 expression is significantly correlated with poor prognosis of CRC patients and mediates the CD8^+^ T cell response in CRC. These findings indicate that lncRNA KCNQ1OT1 is a prognostic biomarker and potential immune therapeutic target for enhancing the CD8^+^ T cell response in CRC.

## Introduction

The occurrence of carcinoma has steadily increased over the past few years, and cancer has become a global health problem. According to WHO estimates, the number of new cancer patients worldwide will reach more than 14 million by 2025 1. Surgery, radiotherapy, and chemotherapy are the main treatment options for tumors, but the prognosis of cancer patients at the advanced stage continues to be unfavorable, especially in some patients with lymph node metastasis 2, 3. Therefore, exploring novel biomarkers and targets is crucial for advanced cancer therapy.

With a length of >200 nucleotides, lncRNAs are non-protein coding transcripts and can regulate gene expression. LncRNAs have been described as “transcriptional noise” and have received little attention in recent decades 4. Increasing evidence reveals that lncRNAs participate in tumorigenesis and development. Thus, lncRNAs may become potential biomarkers and targets for tumor therapy 5. However, only a few lncRNAs have been confirmed to have corresponding functional characteristics, and the functions of most lncRNAs have not been elucidated 6.

Recent studies have indicated that lncRNA KCNQ1OT1 participates in various biological processes, such as cell proliferation, apoptosis, and drug resistance 7-9. In addition, lncRNA KCNQ1OT1 is involved in the progression of different cancers, including breast carcinoma 10, CRC 8, 11, 12, tongue carcinoma 13, lung carcinoma 14, and stomach carcinoma 15. The clinicopathological characteristics, including overall survival, lymphatic meta-stasis, TNM stage, and tumor size, are also positively correlated with lncRNA KCNQ1OT1 expression levels in cancers 16-19. These findings suggest that lncRNA KCNQ1OT1 may be used as a new tumor prognostic biomarker. Nevertheless, because of the limited sample size and discrete outcomes of previous independent studies, there is no consensus on the prognostic value of lncRNA KCNQ1OT1 in cancer patients. Here, we revealed that the expression levels of lncRNA KCNQ1OT1 and CD155 were significantly increased in CRC. In addition, lncRNA KCNQ1OT1 positively correlated with CD155 in CRC. The potential functions and immune response of lncRNA KCNQ1OT1 were also explored by bioinformatics and cell coculture systems.

## Material and methods

### The meta-analysis procedure

Our study aims to analyze the role of lncRNA KCNQ1OT1 in various participants with cancer. The following selection criteria were used: (a) articles that explored the association between lncRNA KCNQ1OT1 expression and cancer prognosis; (b) studies with participants divided into high and low lncRNA KCNQ1OT1 expression groups; (c) articles that described related clinicopathologic parameters such as age, sex, LNM, TNM stage, tumor size and distant metastasis (DM); and (d) studies that included sufficient data for the computation of odds ratios (ORs) and corresponding 95% confidence intervals (CIs). The exclusion criteria were as follows: (a) duplicate publications; (b) reviews, letters, case reports, and nonhuman subject research; and (c) articles without usable data and articles that were not published in English.

Two authors (ZL and PL) searched related keywords in PubMed, Web of Science, Medline, and Embase before February 1, 2021, and the search terms for MESH included “LncRNA KCNQ1OT1”, “Lnc KCNQ1OT1”, “long intergenic non-coding RNA KCNQ1OT1”, “lncRNA”, “non-coding RNA”, “cancer”, “tumor”, “cancer”, “carcinomas”, “prognostic”, “prognosis”, “result”, “outcome”, and “survival”. Any conflict with terms was resolved through group discussions. Two researchers (ZL and PL) independently assessed the quality of this study based on the Newcastle Ottawa Scale (NOS), including selection, comparability, and outcome.

Data extraction was conducted by the authors (ZL and PL) according to the selection and exclusion criteria. Disagreements were resolved with the author (ZZ) in group discussions. Study characteristics, clinical outcomes and pathological characteristics were extracted from the remaining studies. If there was an article explaining the detailed HR and 95% survival CI, survival data were directly applied; if not, HR and 95% CI were extracted from Kaplan-Meier curves through Engauge Digitizer 20.

### Integrative bioinformatics analysis

Gene Expression Profiling Interactive Analysis (GEPIA) is a newly developed interactive web server for the analysis of RNA sequencing expression data from tumors and normal samples from the Cancer Genome Atlas and Genotype Tissue Expression (GTEx) project 21. To validate our results further, we utilized the GEPIA database based on the TCGA dataset to investigate lncRNA KCNQ1OT1 expression levels in various cancers. We obtained the expression information of lncRNA KCNQ1OT1 in carcinoma tissues and paracarcinoma tissues from the TIMER database and related prognosis statistics from the GEPIA database. LnCeVar 22, a comprehensive database of genomic variations, was applied to identify dysregulated ceRNA networks and conduct gene ontology and functional enrichment analyses.

Tumor Immune Estimation Resource (TIMER) 23, a website tool for the analysis of tumor-infiltrating immune cells, was used to analyze the correlation of lncRNA KCNQ1OT1 with the level of primary immune cell infiltration. Then, analysis of the correlation between lncRNA KCNQ1OT1 expression and the levels of tumor-infiltrating immunocyte markers was conducted in the TIMER database. According to the immune cell infiltration level, we established a prognostic model of lncRNA KCNQ1OT1 expression using public datasets from TIMER.

### Human tissue samples

Thirty matched CRC tissues and tumor-adjacent tissue were selected from CRC patients who did not receive chemotherapy or radiotherapy after surgery. The resected samples were immediately stored in a -80 °C liquid nitrogen freezer. In accordance with the Declaration of Helsinki, this study was approved by the Ethics Committee of the First Affiliated Hospital of Jinan University, and all patients signed an informed consent form.

### CD8^+^ T cell isolation and culture

According to the manufacturer's instructions, CD8^+^ T cells were purified directly from whole blood using the EasySep Direct Human CD8^+^ T Cell Isolation Kit (STEMCELL Technologies, Cambridge, MA, USA). HCT116 and SW620 cells were obtained from the American Type Culture Collection (Manassas, VA, USA). Both cell lines were cultured in Dulbecco's modified Eagle's medium (Gibco, New York, USA) supplemented with 10% FBS and 1% PS. The cells were maintained at 37 °C in a humidified atmosphere incubator with 5% CO_2_ for further experiments.

### Cell transfection

Small interfering RNAs (siRNAs) against lncRNA KCNQ1OT1 (si-KCNQ1OT1 #1 sequence: 5'-GGUAGAAUAGUUCUGUCUU-3', si-KCNQ1OT1#2 5'-GCCAAUAGCAAC UGACUAA -3' were purchased from RiboBio (Guangzhou, China). HCT116 and SW620 cells were transfected with Lipofectamine 2000 (Invitrogen, Carlsbad, CA, USA) according to the manufacturer's protocol.

### Cell coculture system

CD8^+^ T cells were sorted and cocultured with HCT116 and SW620 cell lines in regular culture plates at different ratios. The cocultured cells were incubated for two days at 37 °C under 5% CO_2_ and harvested for the following experiments.

### Western blotting

Cell and tissue lysates were centrifuged at 12000×g for 15 min to obtain the supernatants. The prepared sample was electrophoresed through SDS-PAGE electrophoresis gel after quantification and then transferred to a PVDF membrane via the sandwich method. The membranes were blocked with 5% BSA at room temperature and incubated with primary antibodies at 4 °C overnight, followed by HRP-conjugated secondary antibodies for 1 hour. Antibodies against CD155 and β-actin were purchased from Cell Signaling Technology (Beverly, MA, USA). Immunoreactive proteins were detected using an ECL Chemiluminescence Detection Kit.

### Real-time Quantitative PCR

Cells were seeded in 6-well plates. Total RNA was isolated with E.Z.N.A. A Total RNA Kit I was used according to the manufacturer's protocol. Reverse transcription of total RNA was performed using a Transcriptor First Strand cDNA Synthesis Kit (Roche, Mannheim, Germany). RT-qPCR was performed using 2 × SYBR Green PCR Master Mix (Bimake, Huston, TX, USA) and specific primers as follows: CD155 forward primer 5'-GCTAGAAGGACTCACTAGACTCAGGAA-3', reverse primer 5'-GTCGCCTCATCTGTCGTGGAAC-3'; β-actin forward primer 5'-GGCACAGTCAAGGCTGAATG-3', reverse primer 5'-ATGGTGGTGAAGACGCCAGTA-3'; and lncRNA KCNQ1OT1 forward primer 5'-AGGGTGACAGTGTTTCATAGGCT-3', reverse primer 5'GAGGCACATTCATTCGTTGGT-3'.

### Flow cytometric analysis

In brief, isolated CD8^+^ T cells from whole blood were stained with PE-conjugated anti-CD8 antibody, and CRC cell lines were stained with V450-conjugated anti-IFN γ antibody. Coculture samples were analyzed using flow cytometry (FACSCalibur; Thermo Fisher Scientific, Inc.).

### Statistical analysis

The quality of the included articles was evaluated by the Newcastle-Ottawa Scale (NOS) 24. Stata statistical software version 12.0 (Stata Corporation, College Station, TX, USA) and Review Manager version 5.3 (Cochrane Collaboration) were used to evaluate all statistical analyses. The heterogeneities of the selected literature were estimated by card-based q-tests and *I*^2^ statistics 25, 26. Spearman correlation analysis and significance analysis were performed on the correlation of gene expression. GraphPad Prism 6.0 software (GraphPad Software, La Jolla, CA, USA) was used for data analysis. The data presented are the mean values of three independent experiments. Differences between two groups were evaluated through Student's t-test, whereas differences among multiple groups were calculated using ANOVA. A P-value<0.05 was considered statistically significant.

## Results

### Characteristics of eligible studies

A flow diagram of the literature screening and selection processes was created for this study (Fig. S1). A total of 388 studies were identified, and 28 duplicate reports were eliminated. After excluding irrelevant research and nonhuman thematic research and further evaluation, a total of 1154 individuals were included in this research. The main characteristics and Newcastle-Ottawa Scale of eligible studies are presented in Table S1 and Table S2, respectively.

### Association between lncRNA KCNQ1OT1 and the prognosis of cancer patients

**Figure 1:**
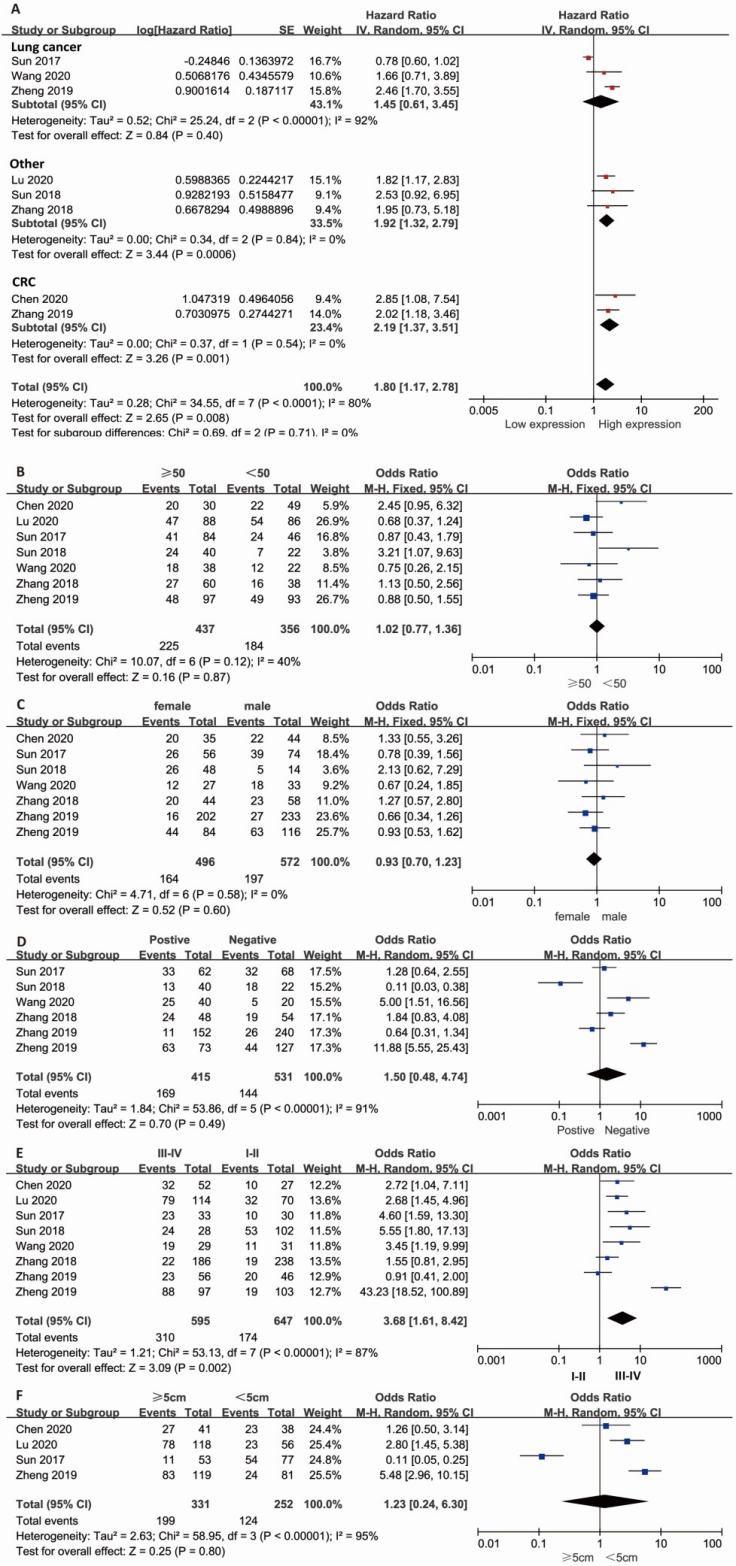
** Meta-analysis of the pooled HRs and clinicopathologic features in various cancers with the expression level of lncRNA KCNQ1OT1.** (A) Pooled HR; (B) Age (>50 vs. <50; (C) Sex (male vs. female); (D) LNM (yes vs. no); (E) TNM stage (III-IV vs. I-II); (F) Tumor size (>5 cm vs. <5 cm).

Combined HR and 95% CI of OS compliance data were collected from 8 studies. The results showed that a high expression level of lncRNA KCNQ1OT1 in cancer tissue correlated with poor OS in patients with overall tumors (pooled HR=1.80, 95% CI: 1.17-2.78; *P*=0.008) (Fig. 1A) with a random-effects model (*I*^2^=80%, *P*<0.001). The subgroup results indicated that high expression of lncRNA KCNQ1OT1 was significantly correlated with poor OS in other types of cancer (pooled HR=1.92, 95% CI: 1.32-2.79, *P*<0.001) and CRC (pooled HR=2.19, 95% CI: 1.37-3.51, *P*=0.001) with low heterogeneity (*I*^2^=0, *P*>0.05). Stratified analyses indicated alteration of the predictive value of lncRNA KCNQ1OT1 for OS in lung cancer (pooled HR=1.45, 95% CI: 0.61-3.45, *P*=0.4). The meta-analysis results indicated that no significant association was detected between lncRNA KCNQ1OT1 expression and age, sex, LNM, or tumor size (Fig. 1B-E). Remarkably, high lncRNA KCNQ1OT1 expression was significantly correlated with advanced TNM stage (OR=3.68, 95% CI: 1.61-8.42,* P*=0.002).

### Subgroup analysis, risk of bias and sensitivity analysis

Because of the significant heterogeneity, we performed a subgroup analysis based on the type of cancer, HR calculation method, cutoff value, sample size and follow-up time with a fixed-effects model. We observed that lncRNA KCNQ1OT1 was a prognostic factor when the HR was obtained though the OS curve (HR = 2.15, 95% CI = 1.67-2.76, *P* < 0.001) but was not significantly associated with OS that was reported directly (*P*>0.05) (Fig. S2B). For the cutoff value of lncRNA KCNQ1OT1, we found that the predictive value was significant only in the median group (HR = 2.13, 95% CI = 1.67-2.71, *P* < 0.001) (Fig. S2D). Then, we found the potential prognostic value of lncRNA KCNQ1OT1 in groups with a sample size <100 patients (HR = 1.97, 95% CI = 1.40-2.77, *P* < 0.001) and a follow-up time ≥60 months (HR = 2.04, 95% CI = 1.39-2.98, *P* < 0.001) with low heterogeneity (*I*^2^=0, *P*>0.1) (Fig. S2A and C).

Asymmetry was observed in the funnel plot (Fig. S3), indicating possible bias of publication, although Egger's test indicated no statistical significance (*Z*=0.45; *P*=0.127) (*P* > 0.05). Moreover, a sensitivity analysis was conducted to assess the influence of each study on the hazard ratio. When the study (Sun et al. 2017) was excluded, the pooled HR was altered considerably (HR = 2.14, 95% CI = 1.72-2.67,* P* < 0.001) and without heterogeneity (*I*^2^=0.0%, *P*=0.93) (Fig. S4). The results indicated that the heterogeneity might come from the study (Sun et al., 2017).

### Functional analysis of lncRNA KCNQ1OT1-related genes across cancers

**Figure 2:**
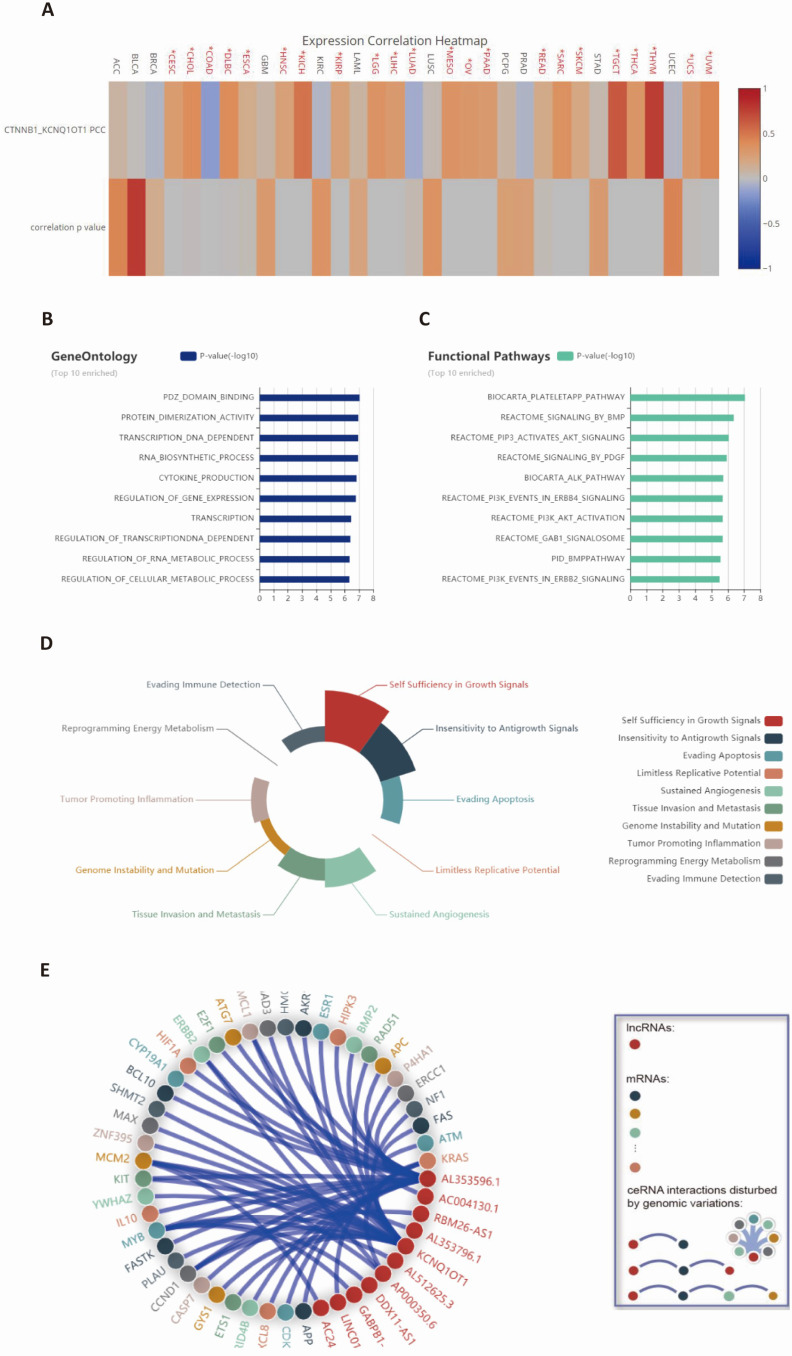
** Functional analysis of lncRNA KCNQ1OT1-related genes across cancers.** (A) Expression correlation heatmap between CTNNB1 and lncRNA KCNQ1OT1 from the LncTarD database (Pearson correlation coefficients). (B-C) Gene ontology and functional pathway enrichment analysis from the LnCeVar database. (D) Hallmark analysis of lncRNA KCNQ1OT1 based on related biological processes. (E) Global view of all possible lncRNA KCNQ1OT1-related ceRNA interactions disturbed by genomic variations.

We curated data from the LncTarD dataset to summarize the potential mechanism and function of lncRNA KCNQ1OT1 in various cancers (Table 2). We observed from the expression correlation heatmap that CTNNB1 was significantly associated with lncRNA KCNQ1OT1 across cancers (Fig. 2A). Integrated analysis showed that gene ontology was mainly involved in PDZ domain binding, protein dimerization activity, and DNA-dependent transcription, and functional pathways were mainly enriched in the biocarta plateletapp pathway and reactome signaling by BMP, and reactome pip3 activates AKT signaling and the biocarta ALK pathway (Fig. 2B-C). Further, we found that lncRNA KCNQ1OT1 participated in dysregulation hallmarks such as self-sufficient growth signals, insensitivity to antigrowth signals, sustained angiogenesis and tumor-promoting inflammation (Fig. 2D). Finally, the network analysis demonstrated a global perspective of potential ceRNA interactions disturbed by genomic variations (Fig. 2E).

### Validation of the results in the TIMER and GEPIA databases

**Figure 3:**
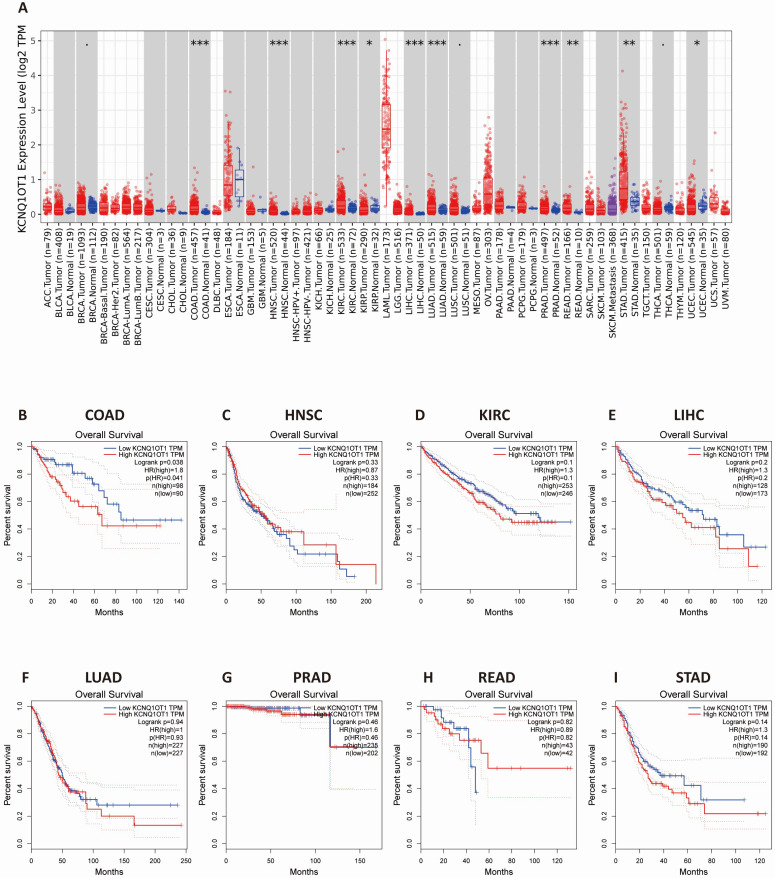
** The expression level and prognostic significance of lncRNA KCNQ1OT1 analyzed by the cancer public database.** (A) LncRNA KCNQ1OT1 expression levels in cancers by the TIMER database. (B-I) Comparison of the survival curves of lncRNA KCNQ1OT1 with high and low expression in different types of tumors in the GEPIA database.

The results from the TIMER database showed that lncRNA KCNQ1OT1 was significantly more highly expressed in colon adenocarcinoma (COAD), head and neck squamous cell carcinoma (HNSC), kidney renal clear cell carcinoma (KIRC), liver hepatocellular carcinoma (LIHC), lung adenocarcinoma (LUAD), prostate adenocarcinoma (PRAD), rectum adenocarcinoma (READ), lung adenocarcinoma, and stomach adenocarcinoma (STAD) than in normal tissues (Fig. 3A). Next, we investigated the relationship between lncRNA KCNQ1OT1 and the survival of different cancer patients in the GEPIA database. Remarkably, high lncRNA KCNQ1OT1 expression was marginally associated with poorer prognosis only in COAD (OS HR = 1.80, log-rank *P* = 0.038) but no significant effect was observed in HNSC, KIRC, LIHC, LUAD, PRAD, READ or STAD (Fig. 3B-I).

### Immune cell infiltration analysis

**Figure 4:**
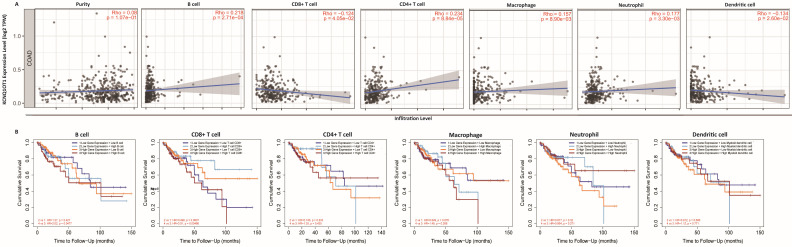
** Immune cell infiltration analysis.** (A) Correlation between lncRNA KCNQ1OT1 expression and immune infiltration in COAD in the TIMER database. (B) Association between immune infiltration level and cumulative survival in COAD with different expression levels of lncRNA KCNQ1OT1.

The above results confirm the prognostic role of lncRNA KCNQ1OT1 and indicate that tumor-infiltrating lymphocytes may influence the survival of patients. Therefore, we explored the association between immune infiltration and lncRNA KCNQ1OT1 expression in patients with COAD. Analysis using the TIMER database showed that lncRNA KCNQ1OT1 expression levels were positively correlated with B cells (*r* = 0.218, *P* = 2.71e-04), CD4^+^ T cells (*r* = 0.234, *P* = 8.84e-05), macrophages (*r* = 0.157, *P* = 8.9e-03), and neutrophils (*r* = 0.177, *P* = 3.30e-03) but negatively correlated with CD8^+^ T cells (*r* = -0.124, *P* = 4.05e-02) and dendritic cells (*r* =-0.134, *P* = 2.60e-02) in COAD (Fig. 4A). We then evaluated the prognostic value of each type of immune cell via Kaplan-Meier analysis. Interestingly, we found that COAD patients with high lncRNA KCNQ1OT1 expression and high CD8^+^ T cell infiltration levels had a worse prognosis than those with low lncRNA KCNQ1OT1 expression and high CD8^+^ T cell infiltration levels (*P* < 0.01) (Fig. 4B). This phenomenon has not been found in other tumors (Fig. S5), suggesting that lncRNA KCNQ1OT1 specially mediates CD8^+^ T cell exhaustion in CRC.

### Relationships between lncRNA KCNQ1OT1 and T cell exhaustion

**Figure 5:**
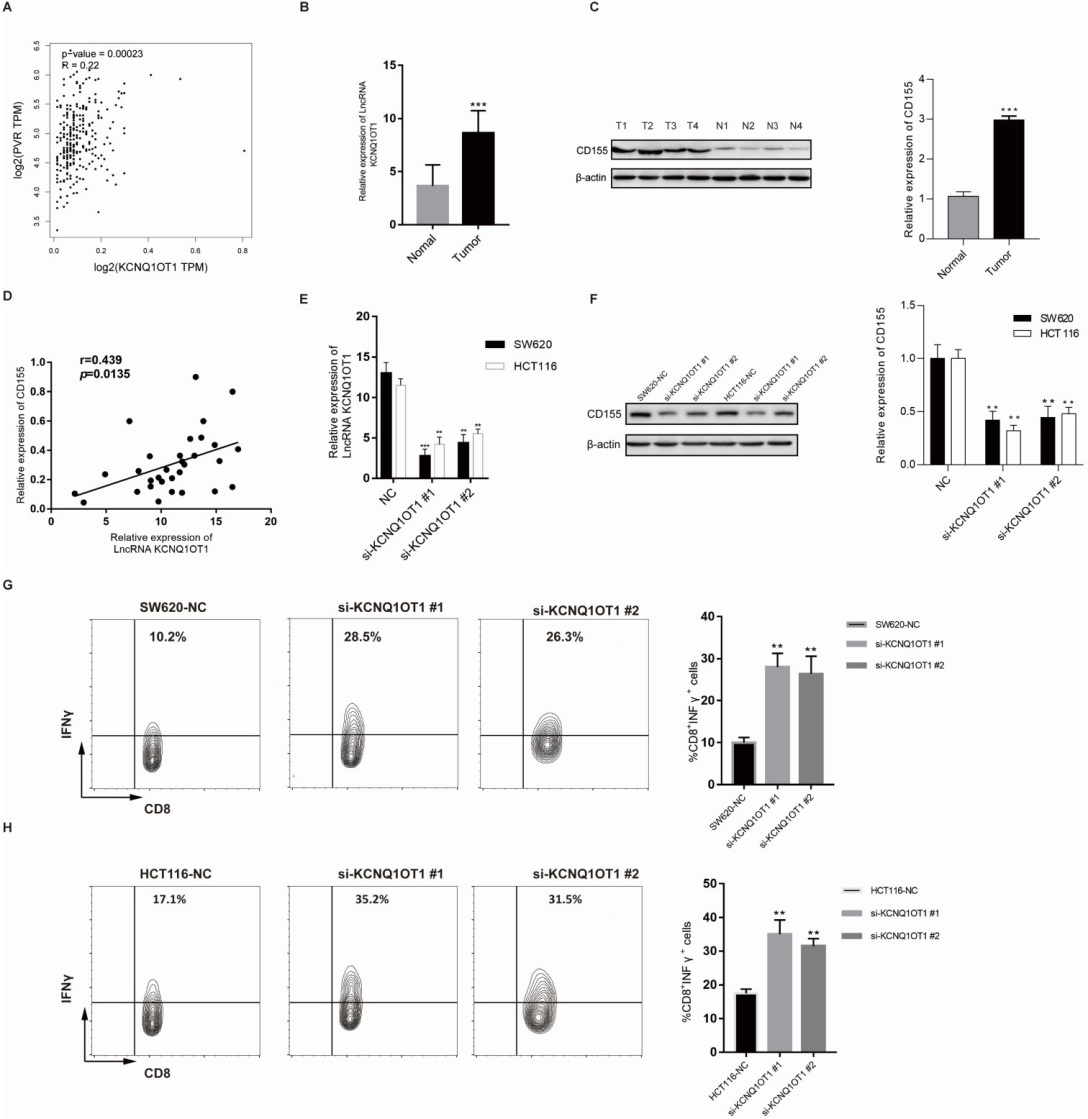
** LncRNA KCNQ1OT1 mediates the CD8^+^ T cell response by regulating CD155 expression in CRC.** (A) Correlation between lncRNA KCNQ1OT1 expression and CD155 in COAD in a public database. (B) The expression of lncRNA KCNQ1OT1 was detected by RT-qPCR in CRC tissues. (C) The expression of CD155 was detected by western blot in CRC tissues. (D) Correlation between lncRNA KCNQ1OT1 expression and CD155 in CRC tissues. (E-F) The expression of lncRNA KCNQ1OT1 and CD155 was detected by RT-qPCR in transfected cells. (G-H) IFN-γ production in CD8^+^ T cells measured by flow cytometry (**P<0.01;***P<0.001).

To further explore the correlation between lncRNA KCNQ1OT1 and immune cell infiltration, we investigated the relationships between lncRNA KCNQ1OT1 and the T cell exhaustion markers CD155 (PVR), PD1, PD-L1 (CD274), TIM-3, and CTLA4 in the TIMER and GEPIA databases. Interestingly, we found significant positive correlations between lncRNA KCNQ1OT1 and CD155 (PVR) in CRC (*r* = 0.22, *P* =0.001) (Table S3, Fig. 5A). CD155 is a crucial protein that regulates CD8^+^ T-cell function by interacting with TIGIT 27-29. Therefore, these results suggested that high lncRNA KCNQ1OT1 expression plays an important role in CD155 mediating the CD8^+^ T cell response.

Next, lncRNA KCNQ1OT1 and CD155 expression was investigated in CRC tissues through RT-qPCR and western blot analysis. Our results showed that lncRNA KCNQ1OT1 and CD155 were significantly more highly expressed in CRC tissue than in adjacent normal tissues (Fig. 5B-C). Moreover, we found that lncRNA KCNQ1OT1 expression positively correlated with CD155 expression levels in CRC tissues (*r*=0.439, *P*=0.0135; Fig. 5D). We knocked down lncRNA KCNQ1OT1 in HCT116 and SW620 cells and found that downregulation of lncRNA KCNQ1OT1 expression inhibited CD155 expression (Fig. 5E-F). Additionally, HCT116 and SW620 cells with silencing of lncRNA KCNQ1OT1 exhibited an enhanced CD8^+^ T cell response in the coculture system (Fig. 5G-H). These data indicated that lncRNA KCNQ1OT1 is crucial for regulating CD155 and mediating the CD8^+^ T cell response in CRC.

## Discussion

LncRNA KCNQ1OT1 is located on chromosome 11p15.5 and plays an essential role in tumor progression 30. LncRNA KCNQ1OT1 participates in tumor proliferation, invasion, metastasis, and antiapoptotic processes and plays a vital role in promoting or suppressing different tumors 30, 31. In colorectal cancer cell lines, lncRNA KCNQ1OT1 induces protective autophagy and chemotherapy resistance by lysing LC3 32. Although many studies highlight the crucial functions of lncRNA KCNQ1OT1, the prognostic value and potential function of lncRNA KCNQ1OT1 in tumors are still controversial. Here, we found that high expression levels of lncRNA KCNQ1OT1 predicted poor OS and advanced TNM stage in CRC. Interestingly, patients with high lncRNA KCNQ1OT1 expression and CD8^+^ T cell infiltration levels had significantly poorer overall survival than patients with low lncRNA KCNQ1OT1 expression and high CD8^+^ T cell infiltration levels. Furthermore, our results revealed that lncRNA KCNQ1OT1 expression levels are positively correlated with CD155 expression. Moreover, inhibiting lncRNA KCNQ1OT1 in CRC cells significantly enhanced the immune response when cocultured with T cells. Therefore, these results indicated that lncRNA KCNQ1OT1 is a prognostic biomarker and novel immune therapeutic target for enhancing the CD8^+^ T cell response in CRC.

First, we performed this meta-analysis with eight studies to confirm the accuracy of previous research results. We found that upregulation of lncRNA KCNQ1OT1 expression was related to poor outcomes and advanced TNM stage in CRC with low heterogeneity. Although our sensitivity analysis revealed that the results only (Sun et al. 2017) impacted the outcomes, the overall heterogeneity was low in our included studies. These results are consistent with the findings of previous studies 12. Indeed, there are several limitations to this study that should be pointed out. First, there were only 8 studies in this meta-analysis, and all the studies were from China. Estimating the HR and 95% CI from the Kaplan-Meier curve may affect the consistency of the results. Second, distinguishing the cutoff value of lncRNA KCNQ1OT1 expression groups may cause heterogeneity. In addition, we used the survival information retrieved by the GEPIA and TIMER databases to further validate our results. The results from the TIMER database showed that lncRNA KCNQ1OT1 was highly expressed in COAD, HNSC, KIRC, LIHC, LUAD, PRAD, READ and STAD compared with normal tissues. Nevertheless, high lncRNA KCNQ1OT1 expression was marginally associated with poorer prognosis only in COAD. The results of the TIMER and GEPIA databases are highly consistent with this meta-analysis.

Our integrated bioinformatics analysis indicated that the most significantly related coexpressed gene of lncRNA KCNQ1OT1 is CTNNB1. Following this observation, the results of gene ontology analysis indicated that lncRNA KCNQ1OT1 was significantly related to PDZ domain binding regulation, protein dimerization activity, and DNA-dependent transcription. Functional pathway annotation demonstrated that lncRNA KCNQ1OT1 was primarily associated with the biocarta plateletapp pathway, and reactome signaling by BMP, and reactome PIP3 activates AKT signaling and the biocarta ALK pathway. Therefore, lncRNA KCNQ1OT1 plays a significant role in the biological process of tumors.

The mechanism by which lncRNA KCNQ1OT1 affects tumors through the regulation of the immune microenvironment is still unclear, so we investigated the role of lncRNA KCNQ1OT1 in tumor immune infiltration in this research. We found correlations between lncRNA KCNQ1OT1 expression levels and tumor prognosis and immune cell infiltration. The expression level of lncRNA KCNQ1OT1 was significantly negatively correlated with CD8^+^ T cell infiltration in patients with COAD. Interestingly, we also observed that high lncRNA KCNQ1OT1 and high CD8^+^ T cell infiltration have a worse prognosis than low lncRNA KCNQ1OT1 and high CD8^+^ T cell infiltration in CRC patients. This result suggests that lncRNA KCNQ1OT1 may affect tumor prognosis through tumor immunity. As one of the key proteins regulating CD8^+^ T cells, CD155 can inhibit the metabolism of CD8^+^ T cells through the TIGIT signaling pathway in gastric cancer 29 and inhibit the immune response by inhibiting the function of CD8^+^ T cell effectors. Therefore, we speculate that lncRNA KCNQ1OT1 may affect tumor immunity by interacting with CD155 to affect the prognosis of tumor patients.

The activation of T cells plays an important role in the antitumor immune response, which relies on activating the AKT/mTOR signaling pathway 33. AKT improves the glucose uptake capacity of T cells by enhancing the expression of glucose transporter 1 34, so AKT/mTOR signaling combines glucose metabolism of T cells with immune response signaling 35. Previous studies have revealed that suspension of AKT and T cell metabolism in the tumor microenvironment can lead to weakening of the antitumor immune response 36, 37. Bioinformatically (Fig. 2C-D), our functional annotation analysis revealed that lncRNA KCNQ1OT1 was enriched in PIP3 activating AKT signaling, while hallmark annotation analysis showed that lncRNA KCNQ1OT1 was significantly associated with tumor promoting inflammation, suggesting that lncRNA KCNQ1OT1 may inhibit T cell immune response by affecting T cell metabolism through the PIP3/AKT pathway. In addition, the upregulated expression of lncRNA KCNQ1OT1 positively correlated with the expression of CD155 in HCT116 and SW620 cell lines. CD155 (PVR/Necl5/Tage4) is a member of the nectin-like adhesion molecule family, which is highly upregulated on tumor cells of many cancer types and is associated with poor patient prognosis 27. CD155 regulates the function of tumor infiltrating lymphocytes by interacting with stimulatory and inhibitory receptors such as TIGIT on T cells and NK cells 38, 39. As a miRNA sponge, lncRNA KCNQ1OT1 can promote immune evasion by upregulating PD-L1 expression in prostate cancer and regulating immune escape in sorafenib-resistant hepatoma carcinoma cell cells 40,41. Furthermore, we found that silencing of lncRNA KCNQ1OT1 downregulated the expression of CD155. Knockdown of lncRNA KCNQ1OT1 in CRC cells promoted IFN-γ production in CD8^+^ T cells, which indicated that the lncRNA KCNQ1OT1 mediates the CD8^+^ T cell response by regulating CD155 in CRC.

## Conclusions

In conclusion, overexpression of lncRNA KCNQ1OT1 is significantly related to malignant prognosis (OS) and clinicopathological features (TNM stage) in different types of cancers, especially CRC. In addition, high lncRNA KCNQ1OT1 expression is significantly correlated with CD155 expression in CRC and mediates CD8^+^ T cell exhaustion in CRC. Therefore, lncRNA KCNQ1OT1 is a prognostic biomarker and potential immune therapeutic target for enhancing the CD8^+^ T cell response in CRC.

## Supplementary Material

Supplementary figures and tables.

## Figures and Tables

**Table 1:** Results of the association between lncRNA KCNQ1OT1 and characteristics of patients with various cancers

Stratified analysis	No. of studies	No. of patients	Pooled HR/OR (95% CI)	*p* value	Heterogeneity		
					I2, %	*p* value	Model
**OS**							
LC	3	390	1.45 (0.61-3.45)	0.404	92	<0.001	Random effects
CRC	2	514	2.19 (1.37-3.51)	0.001	0	0.54	Random effects
Other	3	250	1.92 (1.32-2.79)	<0.001	0	0.84	Random effects
Over all	8	1154	1.80 (1.17-2.78)	0.008	80	<0.001	Random effects
**Clinicopathological features**							
Age (>50 vs. <50)	7	893	1.02 (0.77-1.36)	0.87	40	0.12	Fixed effects
Gender (male vs. female)	7	1068	0.93 (0.70-1.23)	0.60	0	0.58	Fixed effects
LNM (Yes vs. No)	6	946	1.50 (0.48-4.74)	0.49	91	<0.001	Random effects
TNM stage (III-IV vs. I-II)	8	1154	3.68 (1.61-8.42)	0.002	87	<0.001	Random effects
Tumor size (>5 cm vs.<5 cm)	7	583	1.23 (0.24-6.30)	0.80	95	<0.001	Random effects

Abbreviations: CI: confidence interval; HR: hazard ratio; OR: odds ratio; LNM: lymph node metastasis; OS: overall survival; TNM: tumor node metastasis; vs: versus.

**Table 2:** The related regulations of lncRNA KCNQ1OT1 in different cancer

Disease	Regulator	Target	Regulation diretion	Expression parttern	Influenced function	Regulatory mechanism
Colorectal cancer	CTNNB1	KCNQ1OT1	positively-E	upregulation	cancer progression (+)	transcriptional regulation
Breast cancer	KCNQ1OT1	CCNE2	positively-E	upregulation	cell growth (+)	ceRNA (miR-145-5p)
Tongue cancer	KCNQ1OT1	EZR	positively-E	Upregulation	chemoresistance (+); cell proliferation(+); Ezrin/Fak/ Src signaling pathway (-)	ceRNA (miR-211-5p)
Malignant glioma	KCNQ1OT1	CCNE2	positively-E	upregulation	tumor-suppressive function (-)	ceRNA (miR-370)

Abbreviations: positively-E: positively expression.

**Table 3:** Correlation analysis between LncRNA KCNQ1OT1 and relate genes of CD8^+^T cell exhaustion

Gene markers	rho	*p*-value
**CD155(PVR)**	**0.22**	**0.0001**
CD160	0.1145	0.01423
PD1 (PDCD1)	-0.0460	0.3257
PD-L1 (CD274)	-0.1	0.094
CTLA4	0.0207	0.6580
LAG3	-0.0493	0.2928
HAVCR2 (TIM-3)	-0.1309	0.3050

## Abbreviations

LncRNA KCNQ1OT1: Long non-coding RNA KCNQ1 opposite strand/antisense transcript 1
CRC: colorectal cancer
IFN-γ: interferon-gama
NOS: Newcastle-Ottawa Scale
COAD: colon adenocarcinoma
HNSC: head and neck squamous cell carcinoma
KIRC: kidney renal clear cell carcinoma
LIHC: hepatocellular liver carcinoma
LUAD: lung adenocarcinoma
PRAD: prostate adenocarcinoma
READ: rectum adenocarcinoma
STAD: stomach adenocarcinoma
TIGIT: T cell immunoglobulin and ITIM domain
CI: confidence interval
HR: hazard ratio
OR: odds ratio
LNM: lymph node metastasis
OS: overall survival
TNM: tumor node metastasis
