# Supplementary material for: Long Noncoding RNA KCNQ1OT1 is a Prognostic Biomarker and mediates CD8+ T cell exhaustion by regulating CD155 Expression in Colorectal Cancer
Source: Int J Biol Sci. 2021 Apr 22;17(7):1757–68. doi: 10.7150/ijbs.59001 (PMC8120463; doi:10.7150/ijbs.59001)
Supplement: Supplementary file 1 — Supplementary figures and tables. [file ijbsv17p1757s1.pdf]

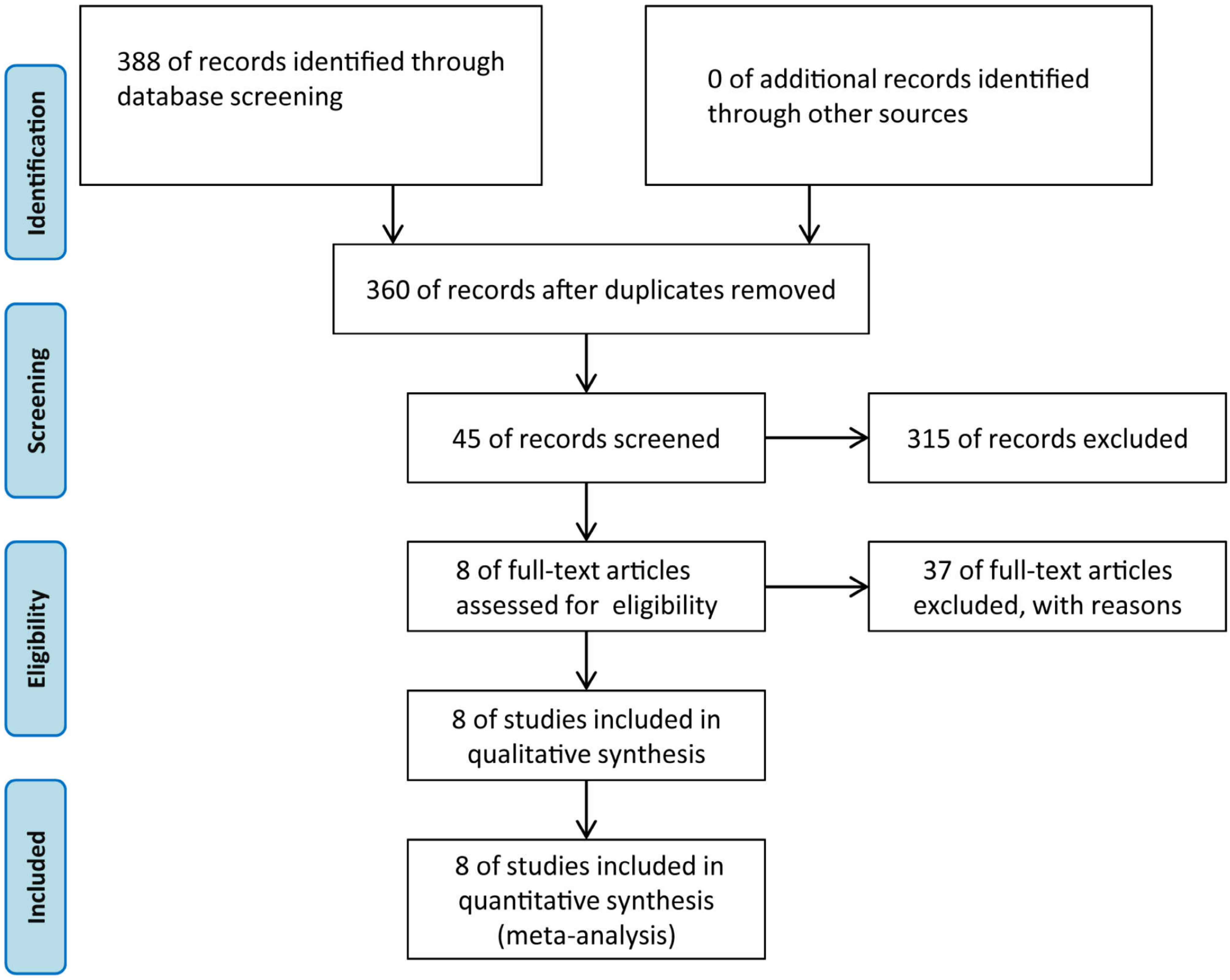
Identification
388 of records identified through database screening
0 of additional records identified through other sources
360 of records after duplicates removed
Screening
45 of records screened
315 of records excluded
Eligibility
8 of full-text articles assessed for eligibility
37 of full-text articles excluded, with reasons
8 of studies included in qualitative synthesis
Included
8 of studies included in quantitative synthesis (meta-analysis)

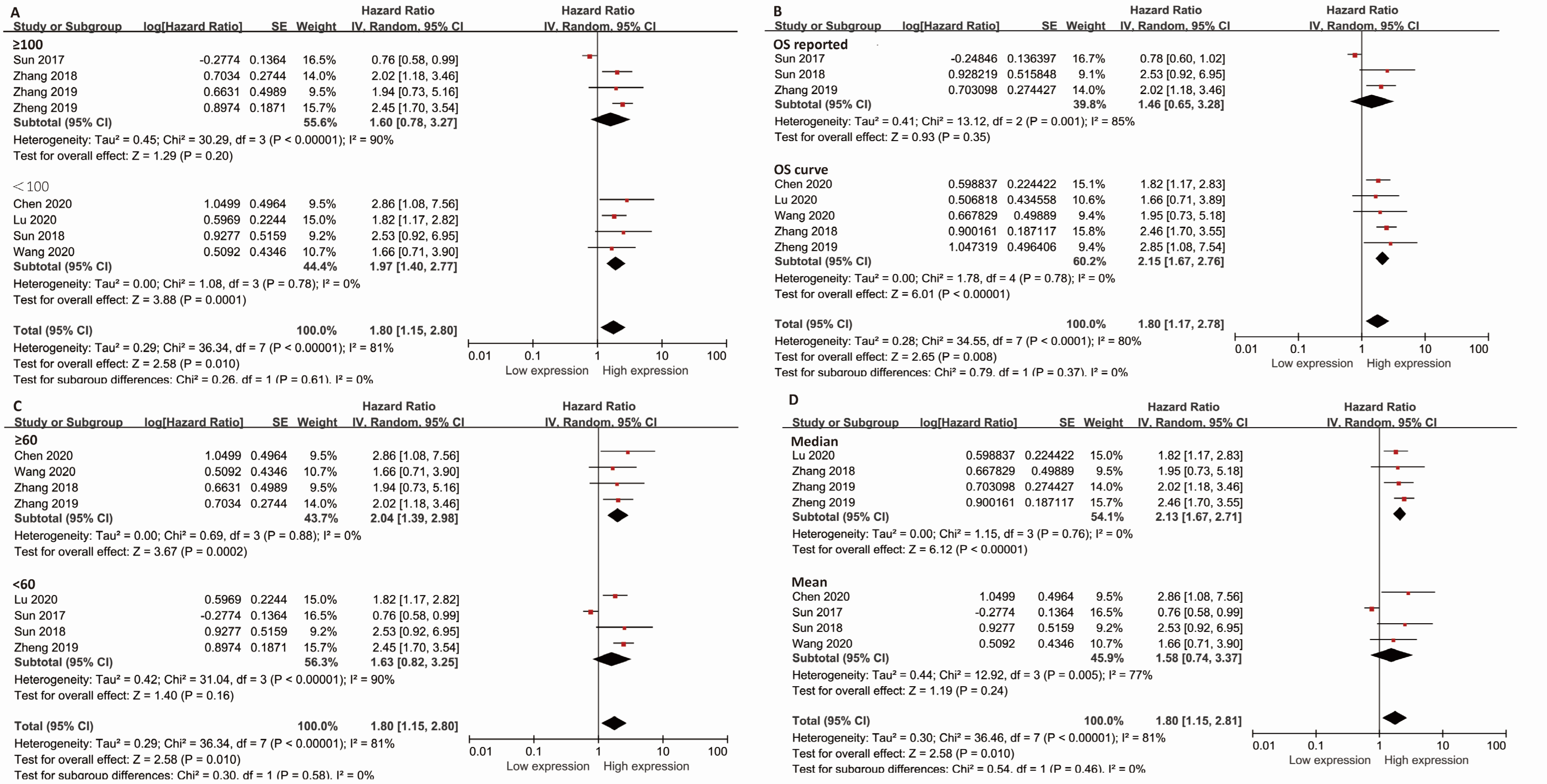
A
Study or Subgroup log[Hazard Ratio] SE Weight Hazard Ratio IV, Random, 95% CI Hazard Ratio IV, Random, 95% CI
≥100
Sun 2017 -0.2774 0.1364 16.5% 0.76 [0.58, 0.99]
Zhang 2018 0.7034 0.2744 14.0% 2.02 [1.18, 3.46]
Zhang 2019 0.6631 0.4989 9.5% 1.94 [0.73, 5.16]
Zheng 2019 0.8974 0.1871 15.7% 2.45 [1.70, 3.54]
Subtotal (95% CI) 55.6% 1.60 [0.78, 3.27]
Heterogeneity: Tau² = 0.45; Chi² = 30.29, df = 3 (P < 0.00001); I² = 90%
Test for overall effect: Z = 1.29 (P = 0.20)
＜100
Chen 2020 1.0499 0.4964 9.5% 2.86 [1.08, 7.56]
Lu 2020 0.5969 0.2244 15.0% 1.82 [1.17, 2.82]
Sun 2018 0.9277 0.5159 9.2% 2.53 [0.92, 6.95]
Wang 2020 0.5092 0.4346 10.7% 1.66 [0.71, 3.90]
Subtotal (95% CI) 44.4% 1.97 [1.40, 2.77]
Heterogeneity: Tau² = 0.00; Chi² = 1.08, df = 3 (P = 0.78); I² = 0%
Test for overall effect: Z = 3.88 (P = 0.0001)
Total (95% CI) 100.0% 1.80 [1.15, 2.80]
Heterogeneity: Tau² = 0.29; Chi² = 36.34, df = 7 (P < 0.00001); I² = 81%
Test for overall effect: Z = 2.58 (P = 0.010)
Test for subgroup differences: Chi² = 0.26, df = 1 (P = 0.61), I² = 0%
0.01 0.1 1 10 100
Low expression High expression
B
Study or Subgroup log[Hazard Ratio] SE Weight Hazard Ratio IV, Random, 95% CI Hazard Ratio IV, Random, 95% CI
OS reported
Sun 2017 -0.24846 0.136397 16.7% 0.78 [0.60, 1.02]
Sun 2018 0.928219 0.515848 9.1% 2.53 [0.92, 6.95]
Zhang 2019 0.703098 0.274427 14.0% 2.02 [1.18, 3.46]
Subtotal (95% CI) 39.8% 1.46 [0.65, 3.28]
Heterogeneity: Tau² = 0.41; Chi² = 13.12, df = 2 (P = 0.001); I² = 85%
Test for overall effect: Z = 0.93 (P = 0.35)
OS curve
Chen 2020 0.598837 0.224422 15.1% 1.82 [1.17, 2.83]
Lu 2020 0.506818 0.434558 10.6% 1.66 [0.71, 3.89]
Wang 2020 0.667829 0.49889 9.4% 1.95 [0.73, 5.18]
Zhang 2018 0.900161 0.187117 15.8% 2.46 [1.70, 3.55]
Zheng 2019 1.047319 0.496406 9.4% 2.85 [1.08, 7.54]
Subtotal (95% CI) 60.2% 2.15 [1.67, 2.76]
Heterogeneity: Tau² = 0.00; Chi² = 1.78, df = 4 (P = 0.78); I² = 0%
Test for overall effect: Z = 6.01 (P < 0.00001)
Total (95% CI) 100.0% 1.80 [1.17, 2.78]
Heterogeneity: Tau² = 0.28; Chi² = 34.55, df = 7 (P < 0.0001); I² = 80%
Test for overall effect: Z = 2.65 (P = 0.008)
Test for subgroup differences: Chi² = 0.79, df = 1 (P = 0.37), I² = 0%
0.01 0.1 1 10 100
Low expression High expression
C
Study or Subgroup log[Hazard Ratio] SE Weight Hazard Ratio IV, Random, 95% CI Hazard Ratio IV, Random, 95% CI
≥60
Chen 2020 1.0499 0.4964 9.5% 2.86 [1.08, 7.56]
Wang 2020 0.5092 0.4346 10.7% 1.66 [0.71, 3.90]
Zhang 2018 0.6631 0.4989 9.5% 1.94 [0.73, 5.16]
Zhang 2019 0.7034 0.2744 14.0% 2.02 [1.18, 3.46]
Subtotal (95% CI) 43.7% 2.04 [1.39, 2.98]
Heterogeneity: Tau² = 0.00; Chi² = 0.69, df = 3 (P = 0.88); I² = 0%
Test for overall effect: Z = 3.67 (P = 0.0002)
<60
Lu 2020 0.5969 0.2244 15.0% 1.82 [1.17, 2.82]
Sun 2017 -0.2774 0.1364 16.5% 0.76 [0.58, 0.99]
Sun 2018 0.9277 0.5159 9.2% 2.53 [0.92, 6.95]
Zheng 2019 0.8974 0.1871 15.7% 2.45 [1.70, 3.54]
Subtotal (95% CI) 56.3% 1.63 [0.82, 3.25]
Heterogeneity: Tau² = 0.42; Chi² = 31.04, df = 3 (P < 0.00001); I² = 90%
Test for overall effect: Z = 1.40 (P = 0.16)
Total (95% CI) 100.0% 1.80 [1.15, 2.80]
Heterogeneity: Tau² = 0.29; Chi² = 36.34, df = 7 (P < 0.00001); I² = 81%
Test for overall effect: Z = 2.58 (P = 0.010)
Test for subgroup differences: Chi² = 0.30, df = 1 (P = 0.58), I² = 0%
0.01 0.1 1 10 100
Low expression High expression
D
Study or Subgroup log[Hazard Ratio] SE Weight Hazard Ratio IV, Random, 95% CI Hazard Ratio IV, Random, 95% CI
Median
Lu 2020 0.598837 0.224422 15.0% 1.82 [1.17, 2.83]
Zhang 2018 0.667829 0.49889 9.5% 1.95 [0.73, 5.18]
Zhang 2019 0.703098 0.274427 14.0% 2.02 [1.18, 3.46]
Zheng 2019 0.900161 0.187117 15.7% 2.46 [1.70, 3.55]
Subtotal (95% CI) 54.1% 2.13 [1.67, 2.71]
Heterogeneity: Tau² = 0.00; Chi² = 1.15, df = 3 (P = 0.76); I² = 0%
Test for overall effect: Z = 6.12 (P < 0.00001)
Mean
Chen 2020 1.0499 0.4964 9.5% 2.86 [1.08, 7.56]
Sun 2017 -0.2774 0.1364 16.5% 0.76 [0.58, 0.99]
Sun 2018 0.9277 0.5159 9.2% 2.53 [0.92, 6.95]
Wang 2020 0.5092 0.4346 10.7% 1.66 [0.71, 3.90]
Subtotal (95% CI) 45.9% 1.58 [0.74, 3.37]
Heterogeneity: Tau² = 0.44; Chi² = 12.92, df = 3 (P = 0.005); I² = 77%
Test for overall effect: Z = 1.19 (P = 0.24)
Total (95% CI) 100.0% 1.80 [1.15, 2.81]
Heterogeneity: Tau² = 0.30; Chi² = 36.46, df = 7 (P < 0.00001); I² = 81%
Test for overall effect: Z = 2.58 (P = 0.010)
Test for subgroup differences: Chi² = 0.54, df = 1 (P = 0.46), I² = 0%
0.01 0.1 1 10 100
Low expression High expression

A

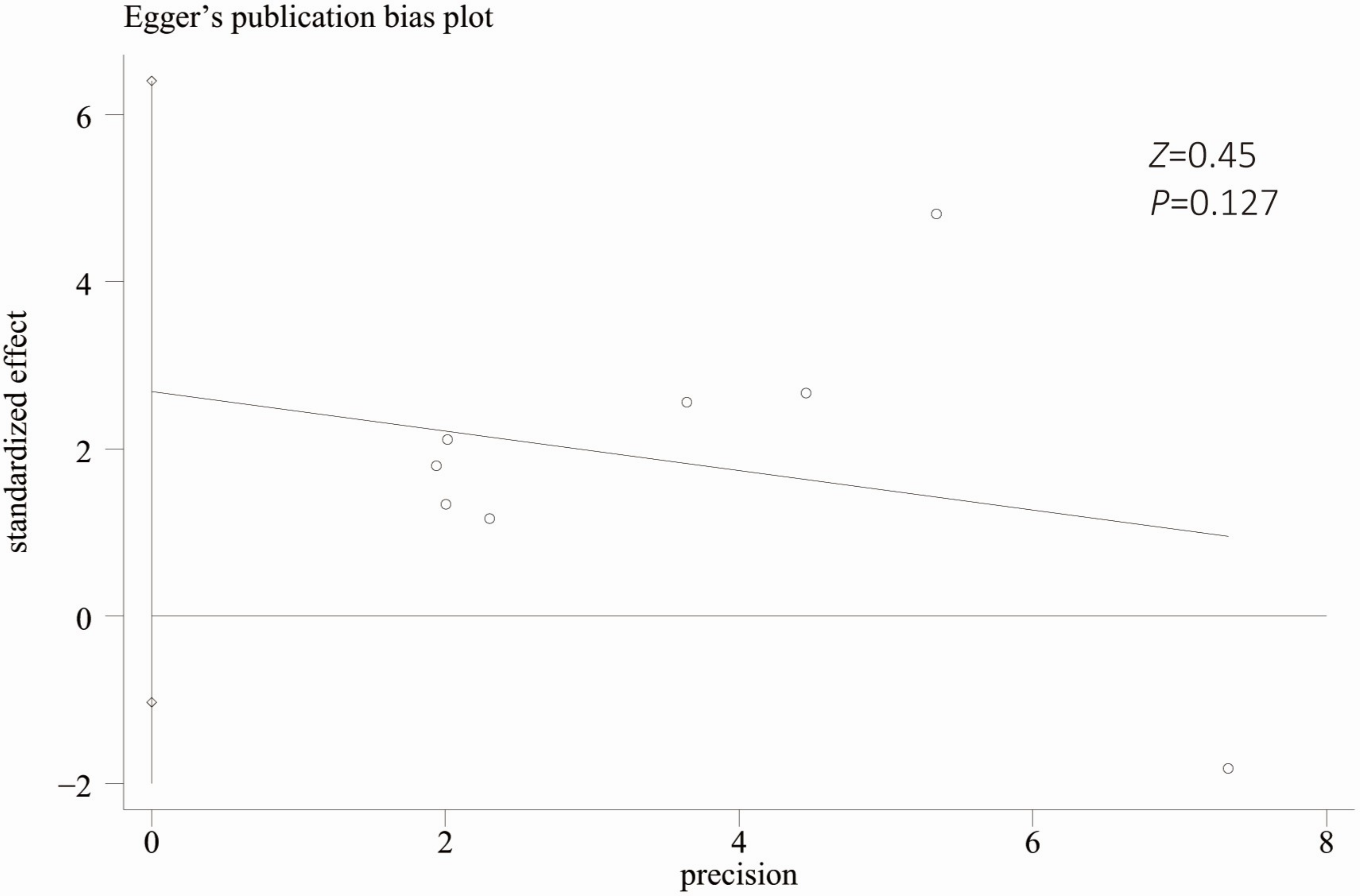

Egger's publication bias plot
Z=0.45
P=0.127
standardized effect
precision
6
4
2
0
−2
0
2
4
6
8

B

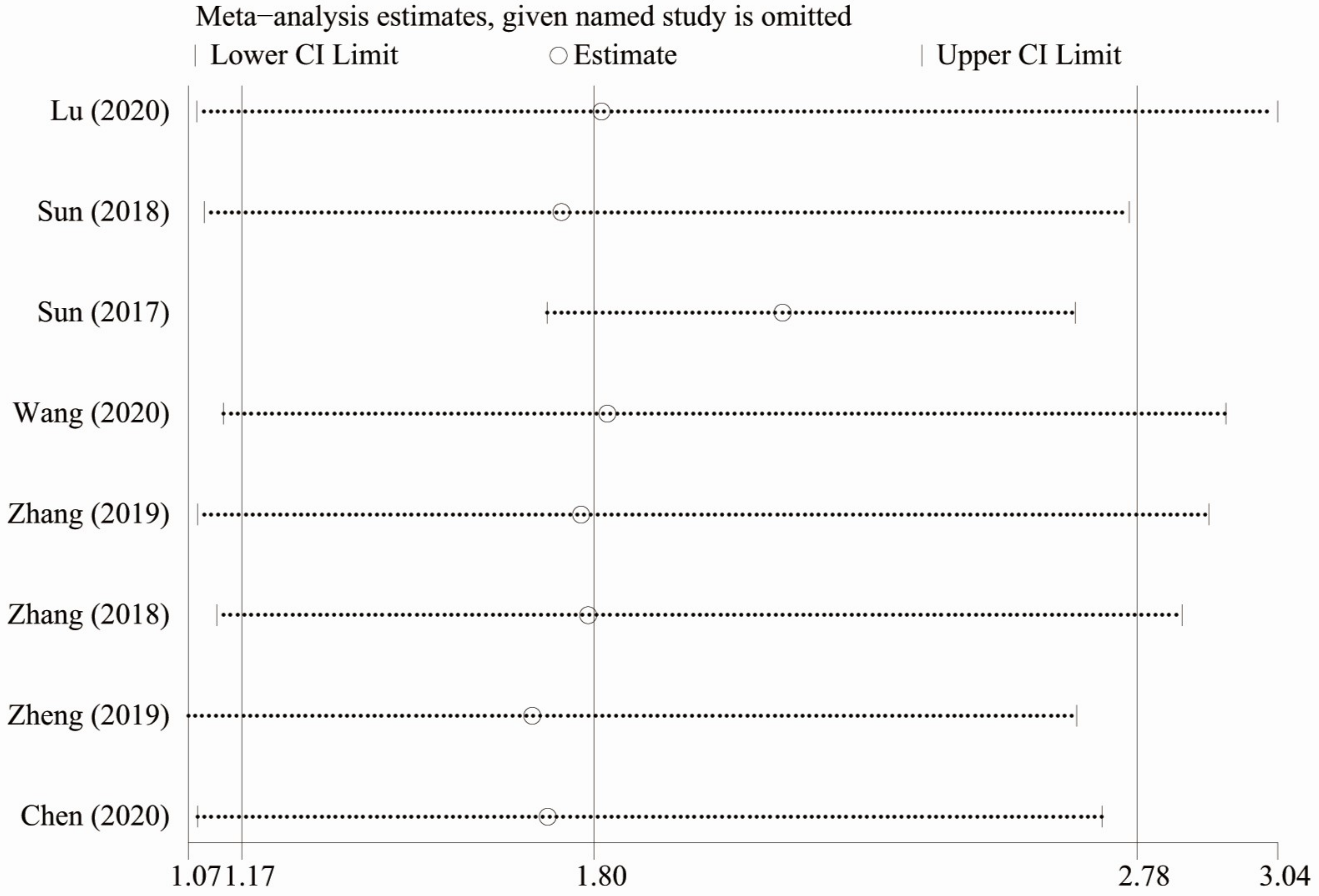

Meta−analysis estimates, given named study is omitted
Lower CI Limit
Estimate
Upper CI Limit
Lu (2020)
Sun (2018)
Sun (2017)
Wang (2020)
Zhang (2019)
Zhang (2018)
Zheng (2019)
Chen (2020)
1.07
1.17
1.80
2.78
3.04

Study or Subgroup	log[Hazard Ratio]	SE	Weight	Hazard Ratio IV, Fixed, 95% CI
**1.8.1 New Subgroup**				
Chen 2020	1.047319	0.4964056	5.1%	2.85 [1.08, 7.54]
Lu 2020	0.5988365	0.2244217	25.2%	1.82 [1.17, 2.83]
Sun 2017	-0.2484614	0.1363972	0.0%	0.78 [0.60, 1.02]
Sun 2018	0.9282193	0.5158477	4.8%	2.53 [0.92, 6.95]
Wang 2020	0.5068176	0.4345579	6.7%	1.66 [0.71, 3.89]
Zhang 2018	0.6678294	0.4988896	5.1%	1.95 [0.73, 5.18]
Zhang 2019	0.7030975	0.2744271	16.8%	2.02 [1.18, 3.46]
Zheng 2019	0.9001614	0.187117	36.2%	2.46 [1.70, 3.55]
**Subtotal (95% CI)**			**100.0%**	**2.14 [1.72, 2.67]**
Heterogeneity: Chi² = 1.93, df = 6 (P = 0.93); I² = 0%				
Test for overall effect: Z = 6.76 (P < 0.00001)				
**Total (95% CI)**			**100.0%**	**2.14 [1.72, 2.67]**
Heterogeneity: Chi² = 1.93, df = 6 (P = 0.93); I² = 0%				
Test for overall effect: Z = 6.76 (P < 0.00001)				
Test for subgroup differences: Not applicable				

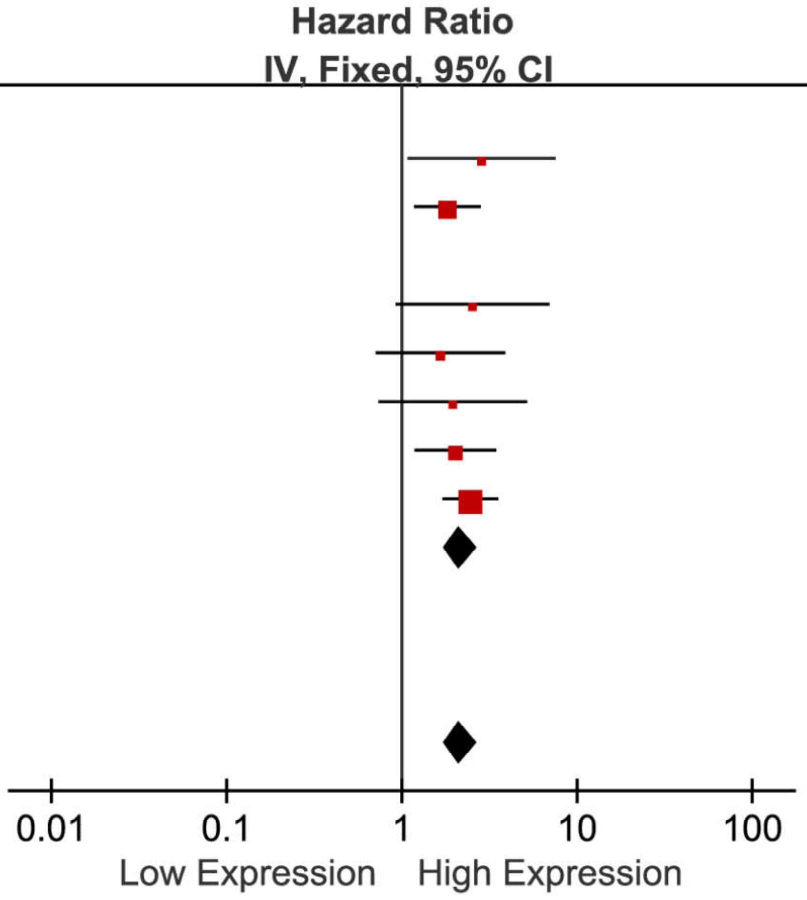

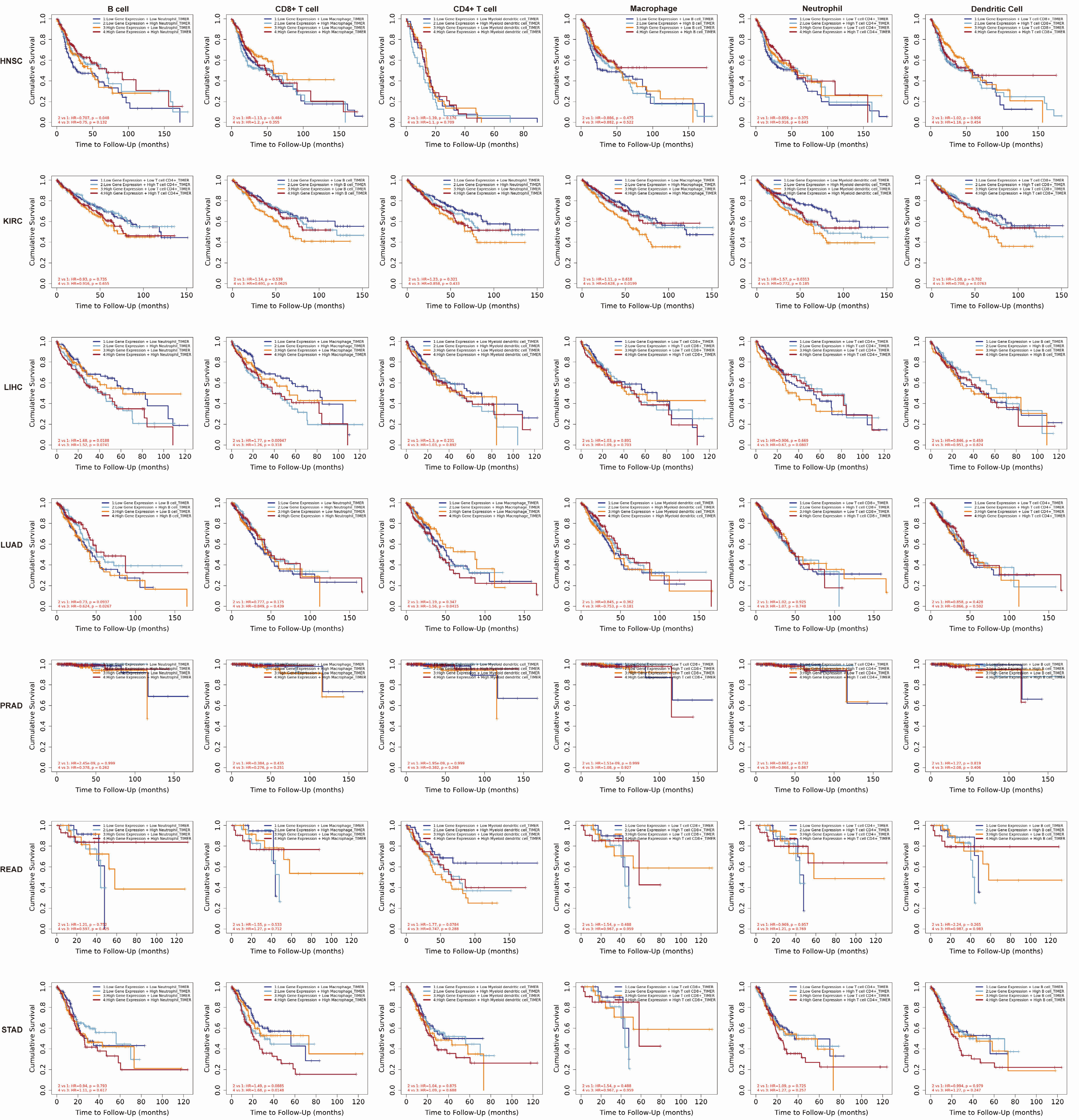
B cell
CD8+ T cell
CD4+ T cell
Macrophage
Neutrophil
Dendritic Cell
HNSC
KIRC
LIHC
LUAD
PRAD
READ
STAD
Cumulative Survival
Time to Follow-Up (months)

Table S1. Characteristics of studies in this meta-analysis

Study	Year	Country	Cancer type	Total	Tumor stage	Method	Cut of	LncRNA KCNQ1OT1 expression				Survival analysis	Multivariate analysis	HR statistic	HR (95% CI)	Follow-up period months	NOS score
								High expression	High with LNM	Low expression	Low with LNM						
Chen[39]	2020	China	CRC	79	I–IV	RT-qPCR	Median	42	NA	37	NA	OS	NA	SC	2.85(1.08-7.56)	80	7
Lu[42]	2020	China	OC	86	I–IV	RT-qPCR	Median	101	NA	73	NA	OS	NA	SC	1.82(1.17-2.82)	60	7
Sun[43]	2018	China	CCA	62	I–IV	RT-qPCR	Mean	31	13	31	27	OS	rep	SC	2.53(0.92-6.95)	57	7
Sun[44]	2017	China	LC	130	I–IV	RT-qPCR	Median	65	33	65	29	OS	rep	SC	0.78(0.58-0.99）	42	6
Wang[19]	2020	China	LC	60	I–IV	RT-qPCR	NA	30	25	30	15	OS	NA	SC	1.66(0.71-3.90)	60	7
Zhang[18]	2019	China	COAD	435	I–IV	RT-qPCR	NA	43	11	392	141	OS	rep	SC	2.02(1.18-3.46）	60	7
Zhang[17]	2018	China	TC	102	I–IV	RT-qPCR	Median	43	24	59	24	OS	NA	SC	1.95(0.73-5.16)	80	6
Zheng[16]	2019	China	LC	200	I–IV	RT-qPCR	Mean	107	63	93	10	OS	NA	SC	2.46(1.70-3.54)	60	7

Abbreviations:

CRC: colorectal cancer; OC: ovarian cancer ;CCA: Cholangiocarcinoma ;LC: Lung cancer; COAD: Colon adenocarcinoma; TC: Tongue cancer; CI: confidence interval; HR: hazard ratio; LNM: lymph node metastasis; NA: no report; OS: overall survival; Rep: report; RT-qPCR: real-time quantitative polymerase chain reaction; SC: survival curve;;BC :breast cancer ;NOS: Newcastle-Ottawa scale

Table S2. **Study quality was assessed according to the Newcastle-Ottawa Scale**

study	selection			comparability	outcome				total
	Adequacy of case definition	Number of case	Representativeness of the cases	Ascertainment of exposure	Ascertainment of detection method	Ascertainment of cut-off	Assessment of outcome	Adequate follow up	
Chen[39]	1	1	1	1	0	1	1	1	7
Lu[42]	1	1	1	0	1	1	1	1	7
Sun[43]	1	1	1	1	1	1	1	0	7
Sun[44]	1	1	1	1	1	1	1	0	6
Wang[19]	1	1	1	0	1	0	1	1	7
Zhang[18]	1	1	1	0	1	0	1	1	7
Zhang[17]	1	1	0	1	1	0	1	0	6
Zheng[16]	1	1	0	1	1	1	1	1	7
